# The structures of 1:1 and 1:2 adducts of phosphane­tricarbo­nitrile with 1,4-di­aza­bicyclo[2.2.2]octa­ne

**DOI:** 10.1107/S2056989021011464

**Published:** 2021-11-16

**Authors:** Andrew P. Purdy, Ray J. Butcher, Christopher A. Klug

**Affiliations:** a Chemistry Division, Code 6100, Naval Research Laboratory, 4555 Overlook Av, SW, Washington DC 20375-5342, USA; b Department of Chemistry, Howard University, 525 College Street NW, Washington DC 20059, USA

**Keywords:** crystal structure, dabco, phosphanetricarbo­nitrile adducts

## Abstract

In the structures of 1:1 and 1:2 adducts of phosphanetricarbo­nitrile (C_3_N_3_P) with 1,4-di­aza­bicyclo­[2.2.2]octane (C_6_H_12_N_2_), the 1:1 adduct crystallizes in the ortho­rhom­bic space group, *Pbcm*, with four formula units in the unit cell (*Z*′ = 0.5). The P(CN)_3_ unit lies on a crystallographic mirror plane while the C_6_H_12_N_2_ unit lies on a crystallographic twofold axis passing through one of the C—C bonds. The P(CN)_3_ moiety has close to *C*_3*v*_ symmetry and is stabilized by forming adducts with two symmetry-related C_6_H_12_N_2_ units. The phospho­rus atom is in a five-coordinate environment. As a result of the symmetry, the two *trans* angles are equal so τ_5_ = 0.00 and thus the geometrical description could be considered to be square pyramidal. However, the electronic geometry is distorted octa­hedral with the lone pair on the phospho­rous occupying the sixth position. As would be expected from VSEPR considerations, the repulsion of the lone-pair electrons with the equatorial bonding electrons means that the *trans* angles for the latter are considerably reduced from 180° to 162.01 (4)°, so the best description of the overall geometry for phospho­rus is distorted square pyramidal. The 1:2 adduct crystallizes in the monoclinic space group, *P*2_1_/*m* with two formula units in the asymmetric unit (*i.e. Z*’ = 1/2). The P(CN)_3_ moiety lies on a mirror plane and one of the two C_6_H_12_N_2_ (dabco) mol­ecules also lies on a mirror plane. The symmetry of the P(CN)_3_ unit is close to *C*_3*v*._ There are three P⋯N inter­actions and consequently the mol­ecular geometry of the phospho­rus atom is distorted octa­hedral. This must mean that the lone pair of electrons on the phospho­rus atom is not sterically active. For the 1:1 adduct, there are weak associations between the phospho­rus atom and one of the terminal nitro­gen atoms from the C≡ N moiety, forming chains in the *a*-axis direction. In addition there are weak C—H⋯N inter­actions between a terminal nitro­gen atoms from the C≡N moiety and the C_6_H_12_N_2_ mol­ecules, which form sheets perpendicular to the *a* axis.

## Abstract 2

The structures of 1:1 and 1:2 adducts of phosphanetricarbo­nitrile with 1,4-di­aza­bicyclo­[2.2.2]octane are reported.

## Chemical context

Phospho­rus tricyanide reacts in solution with nitro­gen bases to produce a large mixture of products. This occurs with dicyan­amides (Epshteyn *et al.*, 2019 ▸), amines, and others. A reaction with CN^−^ was reported to produce an unusual dianion, P_2_C_10_N_10_, which was structurally characterized (Schmidpeter *et al.*, 1985 ▸). However, most of the products from these reactions are unknown. We have followed reactions between tertiary amines and P(CN)_3_ by NMR, which shows many different chemical species as the reaction proceeds, but no crystalline compounds were isolated until P(CN)_3_ was combined with the bidentate amine 4-di­aza­bicyclo­[2.2.2]octane (dabco). From this system we isolated both 1:1 and 1:2 adducts of P(CN)_3_ with dabco. 
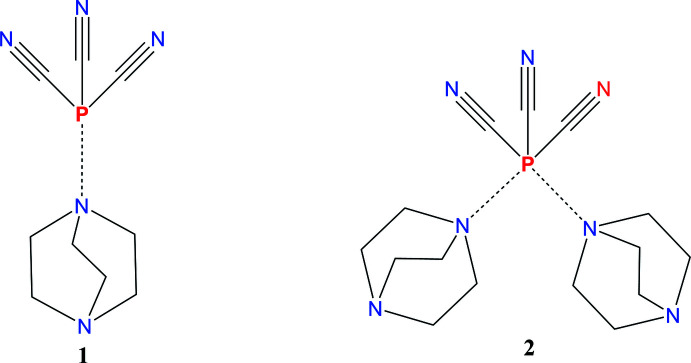


## Structural commentary

**Figure 1:**
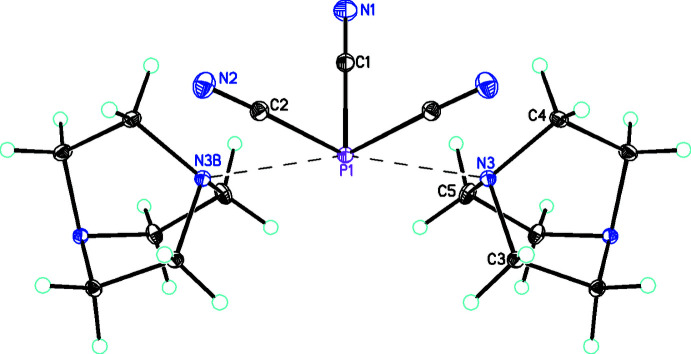
Diagram showing the square-pyramidal coordination sphere of the P atom in **1**. Inter­actions with the C_6_H_12_N_2_ units are shown as dashed bonds. Atomic displacement parameters are at the 30% probability level. The symmetry operation to generate the complete P(CN)_3_ unit is *x*, *y*, 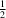 − *z*, and for the complete dabco mol­ecule is *x*, 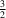 − *y*, 1 − *z*.

**Figure 2:**
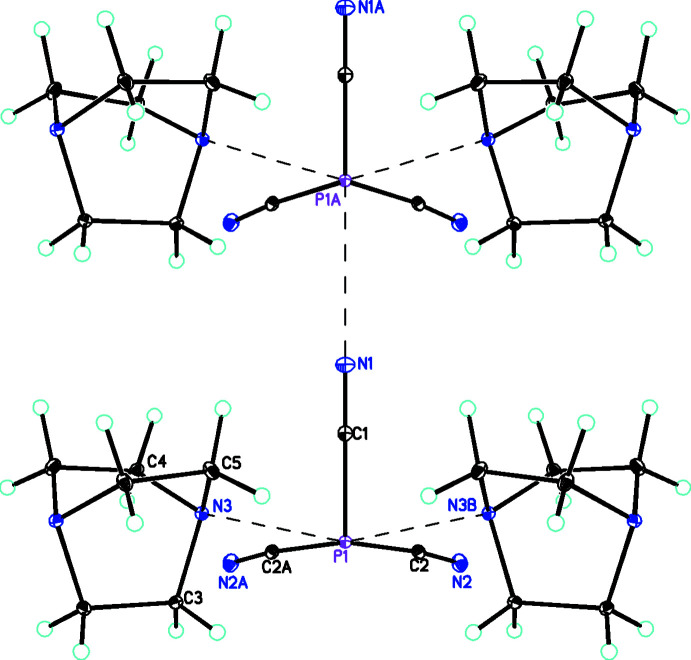
Diagram for **1** showing the inter­action of P1 with N1 (shown as dashed lines), forming chains along the *a-*axis direction. Atomic displacement parameters are at the 30% probability level. The symmetry code for the N1⋯P1*A* inter­action is *x* − 1, *y*, *z*.

The structures of 1:1 (**1**) and 1:2 (**2**) adducts of phosphane­tricarbo­nitrile [P(CN)_3_] with 1,4-di­aza­bicyclo­[2.2.2]octane [C_6_H_12_N_2_] are reported. The 1:1 adduct, P(CN)_3_·(C_6_H_12_N_2_), **1** (Fig. 1 ▸), crystallizes in the ortho­rhom­bic space group, *Pbcm*, with four formula units in the unit cell (*Z*′ = 0.5). The P(CN)_3_ unit lies on a crystallographic mirror plane passing through atoms P1, C1, and N1 while the C_6_H_12_N_2_ unit lies on a crystallographic twofold axis passing through the C3—C3*A* bond. The P(CN)_3_ moiety has close to *C*_3*v*_ symmetry with P—C bond lengths of 1.8057 (15) Å (P1—C1) and 1.8309 (10) Å (P1—C2) and C—P—C bond angles of 87.52 (6)° (C2—P1—C2(*x*, *y*, 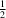 − *z*) and 94.32 (4)° (C1—P1—C2). The P—C≡N bond angles are 174.94 (9)° (P1—C2≡N2) and 176.03 (13)° (P1—C1≡N1). The P(CN)_3_ group is stabilized by forming adducts (Fig. 2 ▸) with two symmetry-related C_6_H_12_N_2_ units of length 2.6562 (8) Å, which is considerably shorter than the sum of their van der Waals radii [P (1.80 Å) + N (1.55 Å) = 3.35 Å; Bondi, 1964 ▸, 1966 ▸]. Including the symmetry-related C_6_H_12_N_2_ and the two P⋯N inter­actions, P1 is in a five-coordinate environment. As a result of the symmetry, the two *trans* angles are equal so τ_5_ = 0.00 (Addison *et al.*, 1984 ▸) so the geometrical description could be considered to be square pyramidal. However, the electronic geometry is distorted octa­hedral with the lone pair on the phospho­rous occupying the sixth position. As would be expected from VSEPR considerations (Gillespie & Nyholm, 1957 ▸; Gillespie, 1970 ▸), the repulsion of the lone-pair electrons with the equatorial bonding electrons means that the *trans* angles for the latter are considerably reduced from 180° to 162.01 (4)°, so the best description of the overall geometry at P1 is distorted square pyramidal. The metrical parameters of the [C_6_H_12_N_2_] units are similar to each other and also show no significant deviations of the metrical parameters of the dabco mol­ecules from values observed in other structures (Szafrański, 2018 ▸; Maderlehner & Pfitzner, 2012 ▸; Goreshnik, 2017 ▸; Akhmad Aznan *et al.*, 2014 ▸).

**Figure 3:**
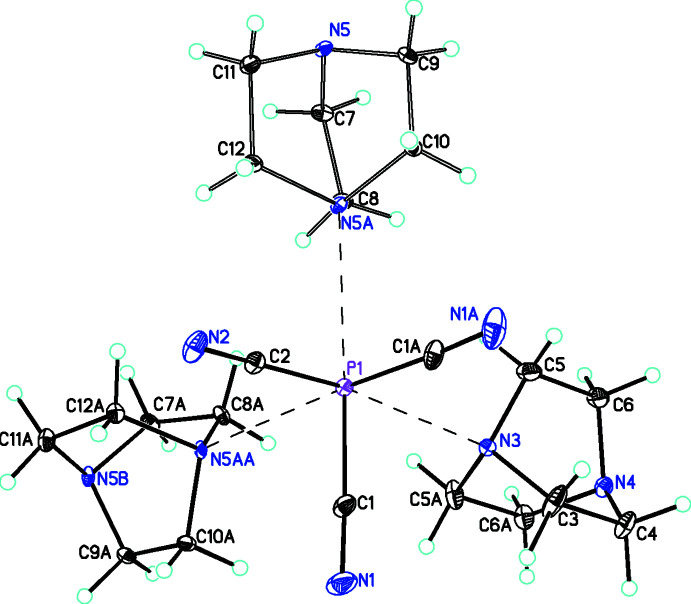
Diagram for **2** showing the distorted octa­hedral coordination geometry of the P atom. Inter­actions with the C_6_H_12_N_2_ units are shown as dashed bonds. Atomic displacement parameters are at the 30% probability level. The symmetry code to generate the P1⋯N5*A* inter­action is 1 − *x*, 1 − *y*, 2 − *z*, and for the P1⋯N5*AA* inter­action is 1 − *x*, *y* − 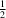 , 2 − *z*.

The second adduct, P(CN)_3_·(C_6_H_12_N_2_), **2** (Fig. 3 ▸), crystallizes in the monoclinic space group, *P*2_1_/*m*, with two formula units in the asymmetric unit (*i.e. Z*’ = 0.5). The P(CN)_3_ moiety lies on a mirror plane passing through atoms P1, C2, and N2 and one of the two C_6_H_12_N_2_ (dabco) mol­ecules also lies on a mirror plane. The symmetry of the P(CN)_3_ unit is close to *C*_3*v*_ with P—C distances of 1.8197 (11) and 1.8315 (15) Å with C—P—C angles of 90.54 (5) and 94.44 (8)°. The PCN groups are almost linear with bond angles of 176.33 (13) and 179.54 (13)°. The P(CN)_3_ group is stabilized by forming asymmetric links to the C_6_H_12_N_2_ units [P1—N3 and P1—N5 distances of 2.6731 (12) and 2.766 (9) Å, respectively]. Both distances are considerably shorter than the sum of their van der Waals radii (Bondi, 1964 ▸, 1966 ▸). Since one of these C_6_H_12_N_2_ units does not lie on a crystallographic symmetry element but P1 does, there are three P⋯N inter­actions and consequently the mol­ecular geometry of P1 is distorted octa­hedral. This must mean that the lone pair of electrons on the P is not sterically active. There is precedence for this in other P^III^ compounds (Capel *et al.*, 2011 ▸).

A comparison of the metrical parameters for the P(CN)_3_ unit of **1** and **2** shows inter­esting differences, in spite of the fact that both lie on mirror planes and thus have the same overall symmetry. In the case of **1**, P1, C1 and N1 lie in the mirror plane while in **2** it is P1, C2 and N2 that are in the mirror plane. In each case, the P—C distances are significantly different between those that are in and out of the mirror plane. For **1**, the P—C(mirror) distance is 1.8057 (15) Å with the other distance at 1.8309 (10) Å, while in the case of **2**, the P—C(mirror) distance is 1.8315 (15) Å with the other distance at 1.8197 (11) Å. This dissimilarity is also shown by the bond angles about the P atoms. In the case of **1**, the smaller angle [87.52 (6)] involves the symmetry-related C≡N groups while in **2** this angle is the larger angle [94.44 (8)°]. This difference between **1** and **2** might be related to the different geometries about the P atoms in the two structures when the inter­actions with the C_6_H_12_N_2_ groups are included. Some important bond parameters (bond lengths and bond angles) for **1** and **2**, respectively, are given in Tables 1 ▸ and 2 ▸.

There are no significant deviations of the metrical parameters of the C_6_H_12_N_2_ mol­ecules in **1** and **2** from values observed in other structures (Szafrański, 2018 ▸; Maderlehner & Pfitzner, 2012 ▸; Goreshnik, 2017 ▸; Akhmad Aznan *et al.*, 2014 ▸). There are very few reports in the literature of structures involving the P(CN)_3_ unit (Dillon *et al.*, 1982 ▸; Sheldrick *et al.*, 1981 ▸; Emerson & Britton, 1964 ▸). In the structure of P(CN)_3_ (Emerson & Britton, 1964 ▸) the P-C– bond lengths are 1.77 (3), 1.79 (3), and 1.80 (3) Å and the P—C—N angles are 93.2 (2), 93.6 (2), and 93.7 (2)°. In this structure, the central P atom makes three non-bonded inter­molecular associations with neighboring terminal N atoms with lengths of 2.85, 2.98, and 2.97 Å and C—N⋯P angles of 116, 122, and 116°. It can be seen that these metrical parameters for both **1** and **2** agree well with those for the parent P(CN)_3_ mol­ecule. The major difference is in the length of the stronger inter­molecular associations with the C_6_H_12_N_2_ units for **1** and **2** at 2.6562 (8) Å for **1**, and 2.6731 (12) and 2.766 (9) Å for **2**, which is much shorter than that observed for P(CN)_3_. In the other structures containing the P(CN)_3_ unit, one contains this unit as a dimer with long P—Br bond lengths forming two *μ*-Br bridges [[P(CN)_3_Br^−^]_2_ (**3**); Sheldrick *et al.*, 1981 ▸], while the other contains an isolated unit forging an association with a chloride anion [P(CN)_3_Cl^−^ (**4**); Dillon *et al.*, 1982 ▸]. In **3**, the phospho­rus atom and one C≡N moiety lie on a mirror plane and the geometry about the P atom is also square pyramidal (τ_5_ = 0.00). The metrical parameters of the P(CN)_3_ unit for **3** are similar to those in **1** and **2**. On the other hand, for **4** there are some significant differences in the metrical parameters of the P(CN)_3_ unit. In this case, the inter­action of the P atom with the Cl atom is much stronger than that with Br in **3** (2.624 *vs* 3.059 Å) and the geometry about P is four-coordinate of the see-saw type. As a consequence, there is more asymmetry in the P—C bond lengths with that *trans* to Cl being 1.916 Å while the other two are 1.781 and 1.785 Å.

## Supra­molecular features

**Figure 4:**
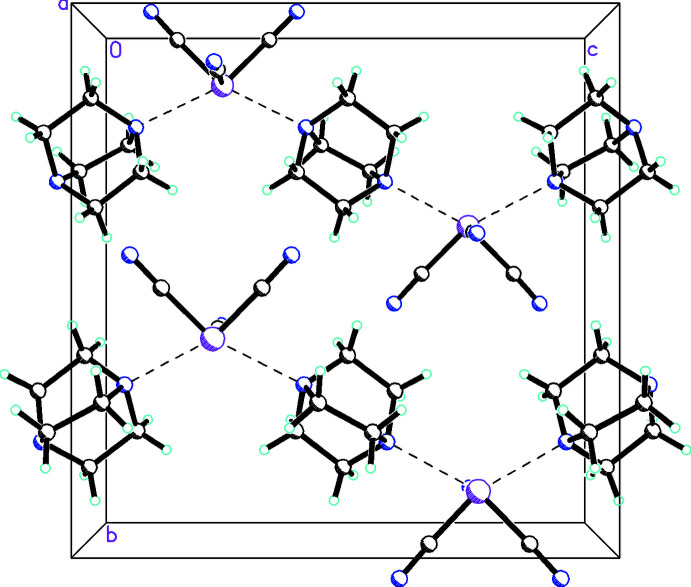
Packing diagram for **1** viewed along the *a* axis. Inter­actions with the C_6_H_12_N_2_ units are shown as dashed bonds.

**Figure 5:**
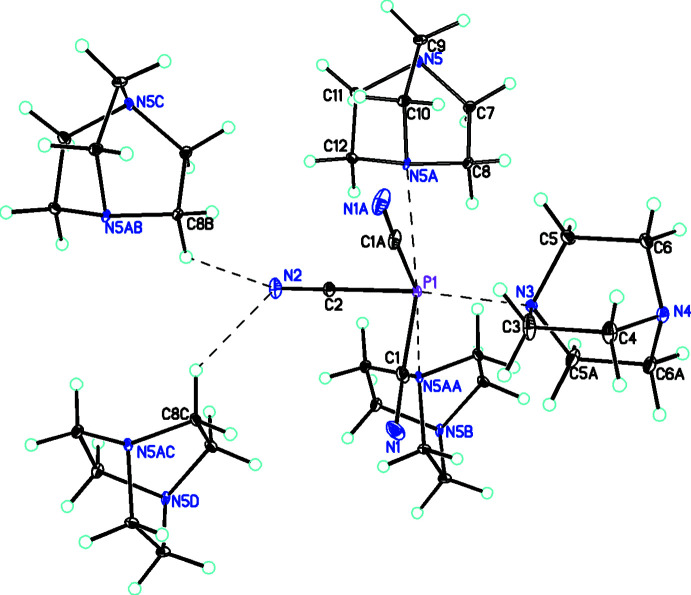
Diagram for **2** showing the bifurcated inter­action of N2 with two C_6_H_12_N_2_ units (shown as dashed bonds). Atomic displacement parameters are at the 30% probability level. The symmetry codes to generate the N2⋯H inter­actions are −*x*, 1 − *y*, 2 − *z*, and −*x*, *y* − 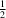 , 2 − *z*.

For **1** there are weak associations between P1 and N1 [3.0806 (14) Å, which, while weak, is shorter than the sum of the van der Waals radii of P and N] from an adjoining P(CN)_3_ unit, forming chains along the *a-*axis direction. In addition there are weak C—H⋯N inter­actions (Table 3 ▸) between N2 and the C_6_H_12_N_2_ mol­ecules, which form sheets perpendicular to the *a* axis (Fig. 4 ▸). For **2**, since the lone pair on P1 is not stereochemically active, there are only weak bifurcated C-H⋯N inter­actions (Table 4 ▸) between N2 and the C_6_H_12_N_2_ mol­ecules, as shown in Fig. 5 ▸.

## Database survey

A search of the Cambridge Structural Database revealed that there are very few reports in the literature of structures involving a P(CN)_3_ unit. The structure of the P(CN)_3_ mol­ecule was published in 1964 (Emerson & Britton, 1964 ▸). There are two other reports of this moiety: one contains this unit as a dimer with long P—Br bond lengths forming two μ-Br bridges (Sheldrick, *et al.*, 1981 ▸), while the other contains an isolated unit forging an association with a chloride anion (Dillon, *et al.*, 1982 ▸). While a majority of reported 1,4-di­aza­bicyclo­[2.2.2]octane (dabco) structures involve these species as protonated cations, dabco is one of the simplest linear bridging ligands that can be used for coordination polymers. There have been several reported examples of dabco-containing coordination polymers, the majority of these also involve another type of bridging ligand or anion (Burrows *et al.*, 2012 ▸; Dau *et al.*, 2012 ▸; Henke *et al.*, 2012 ▸). Unusual examples have been reported where dabco is the sole linking ligand and include one-dimensional (1D) coordination chains (Wang *et al.*, 2011 ▸; Qu & Wu, 2007 ▸; Braga *et al.*, 2004 ▸; Cunha-Silva *et al.*, 2013 ▸), a 2D hexa­gonal network of 6_3_ topology of [Ag(dabco)_3_(H_2_O)]·(3-fluoro­benzene­carboxyl­ate) (Qu & Sun, 2006 ▸) and a series of networks where dabco ligands bridge between *M*_2_I_2_ dimers or between Cu_4_ *X*_4_ or higher order metal clusters where *X* = I or Cl (Shan *et al.*, 2011 ▸; Braga *et al.*, 2010 ▸; Liu *et al.*, 2010 ▸; Zhang *et al.*, 2010 ▸; Bi *et al.*, 2007 ▸; Wiles & Pike, 2006 ▸; O’Keefe *et al.*, 2008 ▸). The latter feature 3D coordination polymer structures with an extraordinary range of topologies. There have also been several cases of metal complexes containing dabco as a ligand, a recent example being {[PMo_8_V_6_O_42_][Cu(dabco)]_2_[Cu(phen)_2_]}·3H_2_O, which exhibits a novel 2D layered framework structure constructed from [PMo_8_V_6_O_42_]^4–^ and two different types of copper complexes (Xiao *et al.*, 2018 ▸).

## Synthesis and crystallization

**General Comments** Phospho­rus cyanide was synthesized from PCl_3_ and 3 eq. of AgCN in CHCl_3_, followed by vacuum sublimation, according to the method of Staats *et al.* (1960 ▸). Aceto­nitrile and chloro­form were dried by distillation from P_2_O_5_ and all reactions were performed in an argon-filled drybox.

**Complexes with dabco**. In an argon-filled dry box, 0.155 g of P(CN)_3_ and 0.35 g of dabco were mixed in a scintillation vial and combined with 15 mL of dry MeCN. The vial was heated with agitation until all solids had dissolved and allowed to cool. The white crystalline product was washed with MeCN and allowed to dry, affording 0.41 g (86%) of the 1:2 adduct (**2**). A reaction performed in a similar manner with 0.24 g of dabco and 0.25 g of P(CN)_3_ produced the 1:1 adduct (**1**), 0.409 g (83%).

**Solid-state NMR**. All solid-state NMR measurements were performed using a Varian 500 spectrometer and a 4 mm HXY triple resonance MAS NMR probe. The ^13^C and ^31^P chemical shifts were referenced using hexa­methyl­benzene and 85% phospho­ric acid, respectively. Rotor-synchronized Hahn-echo pulse sequences with *p*/2 and *p* pulse lengths of 5 ms and 10 ms, respectively, were used to acquire the spectra. Estimates of the spin-lattice relaxation times were obtained by varying the delay between scans. For the extraction of CSA parameters from solid-state spectra, the experimental sideband pattern was compared to an array of sideband patterns and the best match was determined. Final confirmation and an estimate of the error bars was obtained by direct calculation of NMR spectra with the simulation program *SIMPSON* (Bak *et al.*, 2000 ▸).

## Chemical and NMR Discussion

Complexes **1** and **2** have low solubility and only dissociated P(CN)_3_ and dabco were observed by NMR in CD_3_CN or *d*_5_-pyridine solution on a Bruker 400 MHz spectrometer. Other peaks, including P(CN)_2_^−^ (^31^P −194 ppm) and other unidentified species from slow reactions do grow in slowly in a manner similar to solutions of P(CN)_3_ with other amines. Additionally, when a mixture of P(CN)_3_ and 4 eq. of dabco in CD_3_CN was measured, no sharp ^31^P signal for P(CN)_3_ was observed, showing that virtually all the P(CN)_3_ is in the form of insoluble complexes when dabco is present in large excess. However, broad peaks are present in the ^31^P spectrum in all cases where the solids are within the observing region of the NMR spectrometer coil. In order to more fully characterize the complexes by NMR, solid-state magic-angle spinning (MAS) ^31^P and ^13^C NMR spectra were measured on a Varian 500 MHz spectrometer for both **1** and **2**.

**Figure 6:**
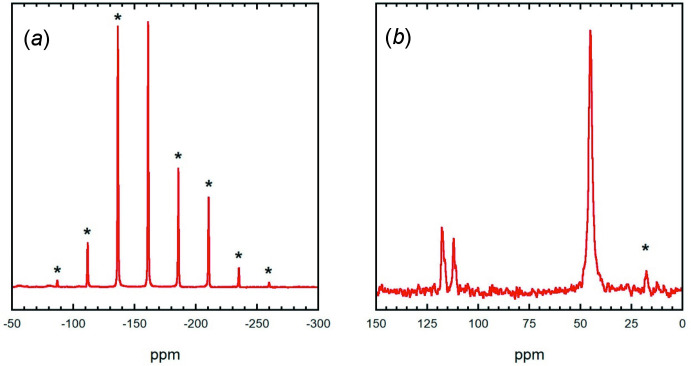
(*a*) ^31^P MAS NMR spectrum for **1** obtained using a spinning speed of 5 kHz. The sideband pattern is corresponds to a chemical shift anisotropy (csa) with isotropic shift of −161 ppm, *d*_aniso_ = −67.7 ppm, and *h* = 0.34; (*b*) ^13^C MAS NMR spectrum for **1** obtained using a spinning speed of 12.5 kHz. Note that in both spectra, spinning sidebands are marked with asterisks (*).

In the native compounds there is only one ^13^C NMR peak for dabco, N(C_2_H_4_)_3_N, located at 47.5 ppm. Phospho­rus cyanide has one peak in both the ^13^C and ^31^P NMR, located at 111.67 ppm and −138.71 ppm, respectively (Chaloux *et al.*, 2015 ▸). The ^31^P and ^13^C NMR spectra for **1** are shown in Fig. 6 ▸. The ^31^P MAS NMR spectrum contains a set of spinning sidebands, which reflect the large chemical shift anisotropy (csa) for this nucleus in **1**. One large peak at 45.1 ppm corresponding to coordinated dabco along with two smaller asymmetric peaks at 112 and 118 ppm in an approximate 1:2 ratio corresponding to nitrile carbons appear in the ^13^C MAS NMR spectrum. This ^13^C NMR spectrum makes sense as there is only one chemically equivalent dabco unit in this structure, but one cyano group has an inter­action with atom P1 of another mol­ecule along *a* (Fig. 2 ▸) and the other two cyano groups do not, making them chemically inequivalent.

**Figure 7:**
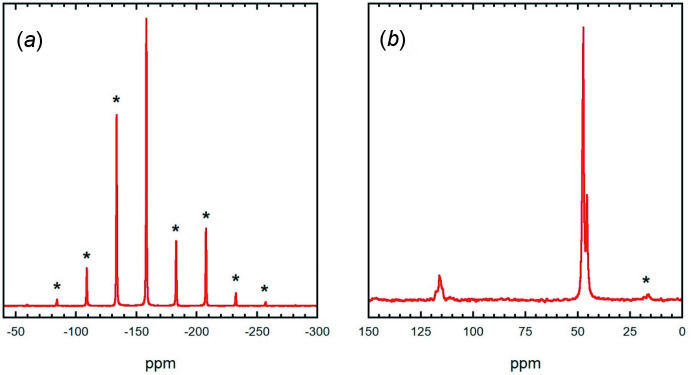
(*a*) ^31^P MAS NMR spectrum for **2** obtained using a spinning speed of 5 kHz. The sideband pattern is corresponds to a chemical shift anisotropy (csa) with isotropic shift of −158 p.p.m., *d*_aniso_ = −59.3 ppm, and *h* = 0.00; (*b*) ^13^C MAS NMR spectrum for **2** obtained using a spinning speed of 12.5 kHz. Note that in both spectra, spinning sidebands are marked with asterisks (*).

The ^31^P and ^13^C NMR spectra for **2** are shown in Fig. 7 ▸. The ^31^P MAS NMR spectrum contains a set of spinning sidebands, which reflect the slightly smaller chemical shift anisotropy (csa) for ^31^P in this compound. Of particular inter­est is that the asymmetry is now close to 0.0, compared to the larger asymmetry of 0.34 for the 1:1 sample, Fig. 6 ▸ *a*. The ^13^C MAS NMR spectrum contains two high field peaks at 47.4 and 45.6 ppm, with the former being roughly three times larger. The peak at 47.4 ppm may correspond to carbon atoms bonded to a dabco nitro­gen that is coordinated to phospho­rus (N5, N3), and the smaller peak to the carbons bonded to N4 that is not coordinated to P1, as these carbons are in a 3:1 ratio. A third asymmetric peak at 116 ppm corresponds to nitrile carbons, which are closer to being chemically equivalent to each other than the nitriles in **1**. Inter­estingly, the spin-lattice relaxation time, *T*_1_, for ^31^P is roughly 10 times shorter for **2** at 45±5 s compared to **1** where a single-exponential fit gives 450±50 s. Similarly, the ^13^C *T*_1_ for the nitrile peak at 116 ppm is 90±10 s for **2**, compared an estimate of 200±50 s for **1**. In both cases the ^13^C *T*_1_ for the low-field peaks near 45 ppm associated with the dabco was much less than 16 s, the shortest delay time used, which makes sense because the dabco units can rotate and are relaxed by their protons. These long ^31^P and cyano spin-lattice relaxation times for **1** are suggestive of a more rigid structure than **2**. The solid-state NMR spectra for both complexes show that they are relatively pure compounds, with little contamination by the other complex.

## Refinement

Crystal data, data collection and structure refinement details are summarized in Table 5 ▸. For both **1** and **2**, all non-hydrogen atoms located from the solution using *SHELXT* (Sheldrick, 2015*a* ▸). Finally, the refinement was completed with anisotropic displacement parameters for all non-hydrogen atoms. The H atoms were located from difference-Fourier maps and constrained to ride on their parent atoms with with C—H bond distances of 0.99 Å and were refined as riding with isotropic displacement parameters 1.2 times that of their C atoms. For **2**, one C_6_H_12_N_2_ unit was located on a symmetry element and its hydrogen atoms were refined isotropically with isotropic displacement parameters 1.2 times that of their C atoms.

## Supplementary Material

Crystal structure: contains datablock(s) 1, 2. DOI: 10.1107/S2056989021011464/yy2005sup1.cif

Structure factors: contains datablock(s) 1. DOI: 10.1107/S2056989021011464/yy20051sup2.hkl

Structure factors: contains datablock(s) 2. DOI: 10.1107/S2056989021011464/yy20052sup3.hkl

Supporting information file. DOI: 10.1107/S2056989021011464/yy20051sup4.cml

Supporting information file. DOI: 10.1107/S2056989021011464/yy20052sup5.cml

CCDC references: 2118658, 2118657

Additional supporting information:  crystallographic information; 3D view; checkCIF report

## Figures and Tables

**Table 1:** Selected geometric parameters (Å, °) for **1** [Chem scheme1]

P1—C1	1.8057 (15)	P1—N3	2.6562 (8)
P1—C2	1.8309 (10)		
			
C1—P1—C2	94.32 (4)	C2—P1—N3	162.01 (4)
C2—P1—C2^i^	87.52 (6)	C2^i^—P1—N3	75.68 (3)
C1—P1—N3	80.83 (3)	N3—P1—N3^i^	120.10 (3)

Symmetry code: (i) x, y, -z+{\script{1\over 2}}.

**Table 2:** Selected geometric parameters (Å, °) for **2** [Chem scheme1]

P1—C1	1.8197 (11)	P1—N3	2.6731 (12)
P1—C2	1.8315 (15)	P1—N5*A*	2.766 (9)
			
C1^i^—P1—C1	94.44 (8)	C1—P1—N5*A*	166.15 (18)
C1—P1—C2	90.54 (5)	C2—P1—N5*A*	78.62 (14)
C1—P1—N3	76.69 (4)	N3—P1—N5*A*	111.45 (11)
C2—P1—N3	160.98 (5)	C1^i^—P1—N5*A* ^i^	166.15 (18)
C1^i^—P1—N5*A*	77.25 (16)	N5*A*—P1—N5*A* ^i^	108.5 (3)

Symmetry code: (i) x, -y+{\script{3\over 2}}, z.

**Table 3:** Hydrogen-bond geometry (Å, °) for **1** [Chem scheme1]

*D*—H⋯*A*	*D*—H	H⋯*A*	*D*⋯*A*	*D*—H⋯*A*
C3—H3*A*⋯N2^ii^	0.99	2.68	3.4269 (13)	133
C3—H3*B*⋯N2^iii^	0.99	2.61	3.3691 (13)	134
C4—H4*B*⋯N2^iv^	0.99	2.64	3.3385 (13)	127

Symmetry codes: (ii) -x+2, y+{\script{1\over 2}}, -z+{\script{1\over 2}}; (iii) -x+2, -y+1, z+{\script{1\over 2}}; (iv) -x+1, -y+1, z+{\script{1\over 2}}.

**Table 4:** Hydrogen-bond geometry (Å, °) for **2** [Chem scheme1]

*D*—H⋯*A*	*D*—H	H⋯*A*	*D*⋯*A*	*D*—H⋯*A*
C8—H8*B*⋯N2^ii^	0.97 (2)	2.53 (2)	3.260 (2)	132 (2)

Symmetry code: (ii) x-1, y, z.

**Table 5:** Experimental details

	**1**	**2**
Crystal data		
Chemical formula	C_6_H_12_N_2_·C_3_N_3_P	2C_6_H_12_N_2_·C_3_N_3_P
*M*_r_	221.21	333.38
Crystal system, space group	Orthorhombic, *P* *b* *c* *m*	Monoclinic, *P*2_1_/*m*
Temperature (K)	105	103
*a*, *b*, *c* (Å)	6.0092 (2), 13.6227 (5), 13.4716 (5)	6.5807 (2), 12.3447 (4), 10.7719 (4)
α, β, γ (°)	90, 90, 90	90, 104.555 (2), 90
*V* (Å^3^)	1102.81 (7)	846.99 (5)
*Z*	4	2
Radiation type	Mo *K*α	Mo *K*α
μ (mm^−1^)	0.23	0.17
Crystal size (mm)	0.30 × 0.20 × 0.04	0.30 × 0.30 × 0.02

Data collection		
Diffractometer	Bruker APEXII CCD	Bruker APEXII CCD
Absorption correction	Multi-scan (*SADABS*; Krause *et al.*, 2015 ▸)	Multi-scan (*SADABS*; Krause *et al.*, 2015 ▸)
*T*_min_, *T*_max_	0.684, 0.746	0.669, 0.747
No. of measured, independent and observed [*I* > 2σ(*I*)] reflections	16377, 1764, 1578	19743, 4235, 3117
*R*_int_	0.035	0.068
(sin θ/λ)_max_ (Å^−1^)	0.716	0.833

Refinement		
*R*[*F* ^2^ > 2σ(*F* ^2^)], *wR*(*F* ^2^), *S*	0.028, 0.068, 1.08	0.049, 0.109, 1.03
No. of reflections	1764	4235
No. of parameters	73	187
No. of restraints	0	18
H-atom treatment	H-atom parameters constrained	H atoms treated by a mixture of independent and constrained refinement
Δρ_max_, Δρ_min_ (e Å^−3^)	0.40, −0.27	0.39, −0.37

Computer programs: *APEX2* (Bruker, 2005 ▸), *SAINT* (Bruker, 2002 ▸), *SHELXT* (Sheldrick 2015*a* ▸), *SHELXL018/3* (Sheldrick, 2015*b* ▸), and *SHELXTL* (Sheldrick, 2008 ▸).

## supplementary crystallographic information

### Phosphanetricarbonitrile–1,4-diazabicyclo[2.2.2]octane (1/1) (1) Crystal data

**Table:**

C_6_H_12_N_2_·C_3_N_3_P	*D*_x_ = 1.332 Mg m^−^^3^
*M**_r_* = 221.21	Mo *K*α radiation, λ = 0.71073 Å
Orthorhombic, *P**b**c**m*	Cell parameters from 7580 reflections
*a* = 6.0092 (2) Å	θ = 3.0–30.5°
*b* = 13.6227 (5) Å	µ = 0.23 mm^−^^1^
*c* = 13.4716 (5) Å	*T* = 105 K
*V* = 1102.81 (7) Å^3^	Plate, colorless
*Z* = 4	0.30 × 0.20 × 0.04 mm
*F*(000) = 464	

### Phosphanetricarbonitrile–1,4-diazabicyclo[2.2.2]octane (1/1) (1) Data collection

**Table:**

Bruker APEXII CCD diffractometer	1578 reflections with *I* > 2σ(*I*)
φ and ω scans	*R*_int_ = 0.035
Absorption correction: multi-scan (SADABS; Krause *et al.*, 2015)	θ_max_ = 30.6°, θ_min_ = 3.0°
*T*_min_ = 0.684, *T*_max_ = 0.746	*h* = −8→8
16377 measured reflections	*k* = −19→19
1764 independent reflections	*l* = −19→19

### Phosphanetricarbonitrile–1,4-diazabicyclo[2.2.2]octane (1/1) (1) Refinement

**Table:**

Refinement on *F*^2^	Primary atom site location: structure-invariant direct methods
Least-squares matrix: full	Secondary atom site location: difference Fourier map
*R*[*F*^2^ > 2σ(*F*^2^)] = 0.028	Hydrogen site location: inferred from neighbouring sites
*wR*(*F*^2^) = 0.068	H-atom parameters constrained
*S* = 1.08	*w* = 1/[σ^2^(*F*_o_^2^) + (0.0261*P*)^2^ + 0.5245*P*] where *P* = (*F*_o_^2^ + 2*F*_c_^2^)/3
1764 reflections	(Δ/σ)_max_ < 0.001
73 parameters	Δρ_max_ = 0.40 e Å^−^^3^
0 restraints	Δρ_min_ = −0.27 e Å^−^^3^

### Phosphanetricarbonitrile–1,4-diazabicyclo[2.2.2]octane (1/1) (1) Special details

**Table:**

Geometry. All esds (except the esd in the dihedral angle between two l.s. planes) are estimated using the full covariance matrix. The cell esds are taken into account individually in the estimation of esds in distances, angles and torsion angles; correlations between esds in cell parameters are only used when they are defined by crystal symmetry. An approximate (isotropic) treatment of cell esds is used for estimating esds involving l.s. planes.

### Phosphanetricarbonitrile–1,4-diazabicyclo[2.2.2]octane (1/1) (1) Fractional atomic coordinates and isotropic or equivalent isotropic displacement parameters (Å^2^)

**Table:**

	*x*	*y*	*z*	*U*_iso_*/*U*_eq_	
P1	0.78467 (6)	0.60830 (2)	0.250000	0.00987 (8)	
N1	0.2954 (2)	0.58880 (10)	0.250000	0.0222 (3)	
N2	0.87850 (16)	0.45325 (6)	0.10017 (7)	0.01964 (18)	
N3	0.69029 (13)	0.69630 (6)	0.42084 (6)	0.01125 (15)	
C1	0.4862 (2)	0.59283 (10)	0.250000	0.0141 (2)	
C2	0.83300 (15)	0.51360 (7)	0.15601 (7)	0.01305 (17)	
C3	0.92037 (15)	0.72302 (7)	0.44943 (6)	0.01195 (17)	
H3A	0.986077	0.766022	0.397947	0.014*	
H3B	1.012485	0.662953	0.454173	0.014*	
C4	0.58489 (16)	0.64329 (7)	0.50448 (7)	0.01500 (18)	
H4A	0.675442	0.585056	0.521857	0.018*	
H4B	0.435121	0.620363	0.484484	0.018*	
C5	0.56488 (17)	0.78818 (7)	0.40371 (7)	0.01575 (19)	
H5A	0.406311	0.772672	0.391407	0.019*	
H5B	0.624346	0.821991	0.344310	0.019*	

### Phosphanetricarbonitrile–1,4-diazabicyclo[2.2.2]octane (1/1) (1) Atomic displacement parameters (Å^2^)

**Table:**

	*U*^11^	*U*^22^	*U*^33^	*U*^12^	*U*^13^	*U*^23^
P1	0.01010 (15)	0.00919 (14)	0.01032 (14)	0.00029 (11)	0.000	0.000
N1	0.0154 (6)	0.0270 (7)	0.0244 (6)	−0.0005 (5)	0.000	0.000
N2	0.0236 (4)	0.0178 (4)	0.0175 (4)	0.0026 (3)	−0.0012 (3)	−0.0025 (3)
N3	0.0108 (3)	0.0119 (3)	0.0110 (3)	−0.0003 (3)	−0.0001 (3)	0.0002 (3)
C1	0.0152 (6)	0.0135 (6)	0.0137 (5)	0.0001 (5)	0.000	0.000
C2	0.0144 (4)	0.0124 (4)	0.0123 (4)	0.0003 (3)	−0.0014 (3)	0.0013 (3)
C3	0.0102 (4)	0.0140 (4)	0.0117 (4)	−0.0007 (3)	0.0009 (3)	−0.0010 (3)
C4	0.0168 (4)	0.0145 (4)	0.0137 (4)	−0.0047 (3)	0.0041 (3)	−0.0015 (3)
C5	0.0170 (4)	0.0160 (4)	0.0142 (4)	0.0045 (3)	−0.0052 (3)	−0.0022 (3)

### Phosphanetricarbonitrile–1,4-diazabicyclo[2.2.2]octane (1/1) (1) Geometric parameters (Å, º)

**Table:**

P1—C1	1.8057 (15)	C3—C3^ii^	1.5481 (17)
P1—C2	1.8309 (10)	C3—H3A	0.9900
P1—C2^i^	1.8309 (10)	C3—H3B	0.9900
P1—N3	2.6562 (8)	C4—C5^ii^	1.5543 (13)
N1—C1	1.148 (2)	C4—H4A	0.9900
N2—C2	1.1474 (13)	C4—H4B	0.9900
N3—C5	1.4792 (12)	C5—H5A	0.9900
N3—C4	1.4807 (12)	C5—H5B	0.9900
N3—C3	1.4807 (12)		
			
C1—P1—C2	94.32 (4)	C3^ii^—C3—H3A	109.6
C1—P1—C2^i^	94.32 (4)	N3—C3—H3B	109.6
C2—P1—C2^i^	87.52 (6)	C3^ii^—C3—H3B	109.6
C1—P1—N3	80.83 (3)	H3A—C3—H3B	108.1
C2—P1—N3	162.01 (4)	N3—C4—C5^ii^	110.23 (7)
C2^i^—P1—N3	75.68 (3)	N3—C4—H4A	109.6
N3—P1—N3^i^	120.10 (3)	C5^ii^—C4—H4A	109.6
C5—N3—C4	108.27 (7)	N3—C4—H4B	109.6
C5—N3—C3	107.95 (7)	C5^ii^—C4—H4B	109.6
C4—N3—C3	108.74 (7)	H4A—C4—H4B	108.1
C5—N3—P1	110.83 (5)	N3—C5—C4^ii^	110.16 (7)
C4—N3—P1	122.05 (5)	N3—C5—H5A	109.6
C3—N3—P1	97.87 (5)	C4^ii^—C5—H5A	109.6
N1—C1—P1	176.03 (13)	N3—C5—H5B	109.6
N2—C2—P1	174.94 (9)	C4^ii^—C5—H5B	109.6
N3—C3—C3^ii^	110.23 (5)	H5A—C5—H5B	108.1
N3—C3—H3A	109.6		
			
C5—N3—C3—C3^ii^	65.44 (11)	P1—N3—C4—C5^ii^	176.24 (6)
C4—N3—C3—C3^ii^	−51.82 (11)	C4—N3—C5—C4^ii^	64.22 (9)
P1—N3—C3—C3^ii^	−179.59 (8)	C3—N3—C5—C4^ii^	−53.34 (10)
C5—N3—C4—C5^ii^	−53.34 (9)	P1—N3—C5—C4^ii^	−159.44 (6)
C3—N3—C4—C5^ii^	63.71 (10)		

Symmetry codes: (i) *x*, *y*, −*z*+1/2; (ii) *x*, −*y*+3/2, −*z*+1.

### Phosphanetricarbonitrile–1,4-diazabicyclo[2.2.2]octane (1/1) (1) Hydrogen-bond geometry (Å, º)

**Table:**

*D*—H···*A*	*D*—H	H···*A*	*D*···*A*	*D*—H···*A*
C3—H3*A*···N2^iii^	0.99	2.68	3.4269 (13)	133
C3—H3*B*···N2^iv^	0.99	2.61	3.3691 (13)	134
C4—H4*B*···N2^v^	0.99	2.64	3.3385 (13)	127

Symmetry codes: (iii) −*x*+2, *y*+1/2, −*z*+1/2; (iv) −*x*+2, −*y*+1, *z*+1/2; (v) −*x*+1, −*y*+1, *z*+1/2.

### Phosphanetricarbonitrile–1,4-diazabicyclo[2.2.2]octane (1/2) (2) Crystal data

**Table:**

2C_6_H_12_N_2_·C_3_N_3_P	*F*(000) = 356
*M**_r_* = 333.38	*D*_x_ = 1.307 Mg m^−^^3^
Monoclinic, *P*2_1_/*m*	Mo *K*α radiation, λ = 0.71073 Å
*a* = 6.5807 (2) Å	Cell parameters from 4473 reflections
*b* = 12.3447 (4) Å	θ = 2.6–35.6°
*c* = 10.7719 (4) Å	µ = 0.17 mm^−^^1^
β = 104.555 (2)°	*T* = 103 K
*V* = 846.99 (5) Å^3^	Plate, colorless
*Z* = 2	0.30 × 0.30 × 0.02 mm

### Phosphanetricarbonitrile–1,4-diazabicyclo[2.2.2]octane (1/2) (2) Data collection

**Table:**

Bruker APEXII CCD diffractometer	3117 reflections with *I* > 2σ(*I*)
φ and ω scans	*R*_int_ = 0.068
Absorption correction: multi-scan (SADABS; Krause *et al.*, 2015)	θ_max_ = 36.3°, θ_min_ = 2.6°
*T*_min_ = 0.669, *T*_max_ = 0.747	*h* = −10→10
19743 measured reflections	*k* = −20→20
4235 independent reflections	*l* = −17→17

### Phosphanetricarbonitrile–1,4-diazabicyclo[2.2.2]octane (1/2) (2) Refinement

**Table:**

Refinement on *F*^2^	Primary atom site location: structure-invariant direct methods
Least-squares matrix: full	Secondary atom site location: difference Fourier map
*R*[*F*^2^ > 2σ(*F*^2^)] = 0.049	Hydrogen site location: mixed
*wR*(*F*^2^) = 0.109	H atoms treated by a mixture of independent and constrained refinement
*S* = 1.03	*w* = 1/[σ^2^(*F*_o_^2^) + (0.0428*P*)^2^ + 0.3013*P*] where *P* = (*F*_o_^2^ + 2*F*_c_^2^)/3
4235 reflections	(Δ/σ)_max_ < 0.001
187 parameters	Δρ_max_ = 0.39 e Å^−^^3^
18 restraints	Δρ_min_ = −0.37 e Å^−^^3^

### Phosphanetricarbonitrile–1,4-diazabicyclo[2.2.2]octane (1/2) (2) Special details

**Table:**

Geometry. All esds (except the esd in the dihedral angle between two l.s. planes) are estimated using the full covariance matrix. The cell esds are taken into account individually in the estimation of esds in distances, angles and torsion angles; correlations between esds in cell parameters are only used when they are defined by crystal symmetry. An approximate (isotropic) treatment of cell esds is used for estimating esds involving l.s. planes.

### Phosphanetricarbonitrile–1,4-diazabicyclo[2.2.2]octane (1/2) (2) Fractional atomic coordinates and isotropic or equivalent isotropic displacement parameters (Å^2^)

**Table:**

	*x*	*y*	*z*	*U*_iso_*/*U*_eq_	Occ. (<1)
P1	0.80141 (5)	0.750000	0.71522 (4)	0.01039 (8)	
N1	1.0735 (2)	0.93032 (11)	0.84218 (11)	0.0353 (3)	
N2	1.0131 (2)	0.750000	0.50118 (14)	0.0236 (3)	
N3	0.74507 (18)	0.750000	0.95248 (11)	0.0113 (2)	
N4	0.6178 (2)	0.750000	1.16108 (12)	0.0163 (2)	
C1	0.97347 (17)	0.85819 (10)	0.79468 (10)	0.0197 (2)	
C2	0.9308 (2)	0.750000	0.58324 (14)	0.0144 (2)	
C3	0.9244 (2)	0.750000	1.06612 (15)	0.0274 (4)	
H3A	1.011990	0.814906	1.064404	0.033*	0.5
H3B	1.011990	0.685094	1.064404	0.033*	0.5
C4	0.8482 (3)	0.750000	1.19066 (15)	0.0228 (3)	
H4A	0.903240	0.685090	1.242177	0.027*	0.5
H4B	0.903240	0.814910	1.242177	0.027*	0.5
C5	0.6170 (2)	0.65328 (10)	0.95859 (12)	0.0249 (3)	
H5A	0.700901	0.587287	0.955230	0.030*	
H5B	0.493677	0.652299	0.883705	0.030*	
C6	0.54197 (19)	0.65328 (10)	1.08391 (11)	0.0208 (2)	
H6A	0.386635	0.651568	1.062498	0.025*	
H6B	0.594804	0.587526	1.134350	0.025*	
N5	0.3804 (14)	0.4230 (7)	0.4470 (5)	0.0112 (8)	0.5
C7	0.2606 (3)	0.50627 (17)	0.4901 (2)	0.0145 (3)	0.5
H7A	0.167 (4)	0.471 (2)	0.533 (3)	0.017*	0.5
H7B	0.174 (4)	0.543 (3)	0.417 (2)	0.017*	0.5
C8	0.4094 (3)	0.58402 (16)	0.5834 (2)	0.0133 (3)	0.5
H8A	0.400 (7)	0.574 (3)	0.670 (2)	0.016*	0.5
H8B	0.369 (4)	0.6579 (15)	0.560 (2)	0.016*	0.5
C9	0.5267 (3)	0.37247 (16)	0.5606 (2)	0.0146 (3)	0.5
H9A	0.441 (5)	0.344 (3)	0.612 (3)	0.017*	0.5
H9B	0.603 (4)	0.3139 (19)	0.535 (3)	0.017*	0.5
C10	0.6913 (3)	0.45691 (16)	0.6299 (2)	0.0134 (3)	0.5
H10A	0.827 (3)	0.441 (3)	0.618 (3)	0.016*	0.5
H10B	0.698 (4)	0.457 (2)	0.7198 (17)	0.016*	0.5
C11	0.5118 (3)	0.47282 (18)	0.3681 (2)	0.0158 (4)	0.5
H11A	0.419 (4)	0.496 (2)	0.288 (2)	0.019*	0.5
H11B	0.604 (7)	0.419 (3)	0.351 (3)	0.019*	0.5
C12	0.6392 (3)	0.56829 (16)	0.44242 (19)	0.0136 (3)	0.5
H12A	0.585 (5)	0.634 (2)	0.400 (4)	0.016*	0.5
H12B	0.784 (3)	0.562 (2)	0.439 (3)	0.016*	0.5
N5A	0.6268 (14)	0.5681 (7)	0.5797 (5)	0.0104 (7)	0.5

### Phosphanetricarbonitrile–1,4-diazabicyclo[2.2.2]octane (1/2) (2) Atomic displacement parameters (Å^2^)

**Table:**

	*U*^11^	*U*^22^	*U*^33^	*U*^12^	*U*^13^	*U*^23^
P1	0.00873 (14)	0.01193 (15)	0.01113 (15)	0.000	0.00363 (11)	0.000
N1	0.0391 (6)	0.0460 (7)	0.0229 (5)	−0.0268 (6)	0.0117 (5)	−0.0107 (5)
N2	0.0148 (6)	0.0415 (9)	0.0151 (6)	0.000	0.0048 (5)	0.000
N3	0.0105 (5)	0.0124 (5)	0.0111 (5)	0.000	0.0028 (4)	0.000
N4	0.0182 (6)	0.0200 (6)	0.0123 (5)	0.000	0.0068 (4)	0.000
C1	0.0182 (5)	0.0289 (6)	0.0134 (4)	−0.0086 (4)	0.0062 (4)	−0.0034 (4)
C2	0.0101 (5)	0.0192 (6)	0.0133 (6)	0.000	0.0019 (4)	0.000
C3	0.0113 (6)	0.0586 (13)	0.0122 (6)	0.000	0.0025 (5)	0.000
C4	0.0183 (7)	0.0388 (10)	0.0107 (6)	0.000	0.0023 (5)	0.000
C5	0.0378 (7)	0.0219 (5)	0.0203 (5)	−0.0148 (5)	0.0172 (5)	−0.0074 (4)
C6	0.0256 (5)	0.0212 (5)	0.0191 (5)	−0.0079 (4)	0.0122 (4)	−0.0017 (4)
N5	0.0117 (11)	0.0142 (16)	0.010 (2)	−0.0046 (10)	0.0063 (16)	−0.0035 (16)
C7	0.0102 (7)	0.0128 (8)	0.0206 (9)	0.0012 (6)	0.0038 (7)	−0.0027 (7)
C8	0.0124 (8)	0.0118 (8)	0.0175 (9)	−0.0001 (6)	0.0072 (7)	−0.0040 (7)
C9	0.0182 (9)	0.0082 (7)	0.0170 (9)	0.0005 (6)	0.0039 (7)	0.0002 (6)
C10	0.0133 (8)	0.0107 (8)	0.0152 (8)	0.0019 (6)	0.0018 (6)	0.0011 (6)
C11	0.0179 (9)	0.0173 (9)	0.0138 (8)	−0.0059 (7)	0.0066 (7)	−0.0033 (7)
C12	0.0148 (8)	0.0125 (8)	0.0146 (8)	−0.0038 (6)	0.0060 (7)	−0.0013 (7)
N5A	0.0134 (11)	0.0121 (14)	0.0084 (19)	0.0008 (9)	0.0077 (16)	−0.0021 (15)

### Phosphanetricarbonitrile–1,4-diazabicyclo[2.2.2]octane (1/2) (2) Geometric parameters (Å, º)

**Table:**

P1—C1^i^	1.8197 (11)	C6—H6B	0.9900
P1—C1	1.8197 (11)	N5—C7	1.441 (9)
P1—C2	1.8315 (15)	N5—C11	1.489 (7)
P1—N3	2.6731 (12)	N5—C9	1.490 (7)
P1—N5A	2.766 (9)	C7—C8	1.548 (3)
P1—N5A^i^	2.766 (9)	C7—H7A	0.967 (16)
N1—C1	1.1484 (16)	C7—H7B	0.962 (16)
N2—C2	1.147 (2)	C8—N5A	1.455 (9)
N3—C3	1.4713 (19)	C8—H8A	0.957 (17)
N3—C5^i^	1.4725 (13)	C8—H8B	0.966 (16)
N3—C5	1.4726 (13)	C9—C10	1.553 (3)
N4—C6^i^	1.4687 (14)	C9—H9A	0.949 (17)
N4—C6	1.4687 (14)	C9—H9B	0.958 (16)
N4—C4	1.469 (2)	C10—N5A	1.497 (8)
C3—C4	1.546 (2)	C10—H10A	0.952 (17)
C3—H3A	0.9900	C10—H10B	0.958 (16)
C3—H3B	0.9900	C11—C12	1.548 (3)
C4—H4A	0.9900	C11—H11A	0.964 (16)
C4—H4B	0.9900	C11—H11B	0.955 (18)
C5—C6	1.5494 (15)	C12—N5A	1.501 (5)
C5—H5A	0.9900	C12—H12A	0.958 (16)
C5—H5B	0.9900	C12—H12B	0.965 (16)
C6—H6A	0.9900		
			
C1^i^—P1—C1	94.44 (8)	C5—C6—H6B	109.5
C1^i^—P1—C2	90.54 (5)	H6A—C6—H6B	108.1
C1—P1—C2	90.54 (5)	C7—N5—C11	109.4 (5)
C1^i^—P1—N3	76.69 (4)	C7—N5—C9	109.1 (4)
C1—P1—N3	76.69 (4)	C11—N5—C9	107.1 (5)
C2—P1—N3	160.98 (5)	N5—C7—C8	110.1 (4)
C1^i^—P1—N5A	77.25 (16)	N5—C7—H7A	107.3 (19)
C1—P1—N5A	166.15 (18)	C8—C7—H7A	110.3 (19)
C2—P1—N5A	78.62 (14)	N5—C7—H7B	110 (2)
N3—P1—N5A	111.45 (11)	C8—C7—H7B	113 (2)
C1^i^—P1—N5A^i^	166.15 (18)	H7A—C7—H7B	106.6 (19)
C1—P1—N5A^i^	77.25 (16)	N5A—C8—C7	111.4 (4)
C2—P1—N5A^i^	78.62 (14)	N5A—C8—H8A	108 (3)
N3—P1—N5A^i^	111.45 (11)	C7—C8—H8A	112 (3)
N5A—P1—N5A^i^	108.5 (3)	N5A—C8—H8B	109.4 (17)
C3—N3—C5^i^	108.14 (8)	C7—C8—H8B	109.1 (17)
C3—N3—C5	108.14 (8)	H8A—C8—H8B	107 (2)
C5^i^—N3—C5	108.35 (13)	N5—C9—C10	110.0 (4)
C3—N3—P1	121.36 (9)	N5—C9—H9A	106 (2)
C5^i^—N3—P1	105.14 (7)	C10—C9—H9A	114 (3)
C5—N3—P1	105.14 (7)	N5—C9—H9B	110.8 (17)
C6^i^—N4—C6	108.77 (13)	C10—C9—H9B	107.1 (17)
C6^i^—N4—C4	107.95 (8)	H9A—C9—H9B	109 (2)
C6—N4—C4	107.95 (8)	N5A—C10—C9	110.0 (4)
N1—C1—P1	176.33 (13)	N5A—C10—H10A	109 (2)
N2—C2—P1	179.54 (13)	C9—C10—H10A	111 (2)
N3—C3—C4	110.79 (12)	N5A—C10—H10B	107.9 (18)
N3—C3—H3A	109.5	C9—C10—H10B	109.5 (17)
C4—C3—H3A	109.5	H10A—C10—H10B	109 (2)
N3—C3—H3B	109.5	N5—C11—C12	109.6 (3)
C4—C3—H3B	109.5	N5—C11—H11A	107.7 (18)
H3A—C3—H3B	108.1	C12—C11—H11A	112.0 (17)
N4—C4—C3	110.72 (12)	N5—C11—H11B	108 (3)
N4—C4—H4A	109.5	C12—C11—H11B	110 (3)
C3—C4—H4A	109.5	H11A—C11—H11B	109 (2)
N4—C4—H4B	109.5	N5A—C12—C11	110.6 (4)
C3—C4—H4B	109.5	N5A—C12—H12A	111 (2)
H4A—C4—H4B	108.1	C11—C12—H12A	108 (3)
N3—C5—C6	110.50 (9)	N5A—C12—H12B	109.8 (19)
N3—C5—H5A	109.6	C11—C12—H12B	109.4 (19)
C6—C5—H5A	109.6	H12A—C12—H12B	107 (2)
N3—C5—H5B	109.6	C8—N5A—C10	107.7 (5)
C6—C5—H5B	109.6	C8—N5A—C12	108.9 (5)
H5A—C5—H5B	108.1	C10—N5A—C12	106.1 (5)
N4—C6—C5	110.84 (9)	C8—N5A—P1	99.0 (4)
N4—C6—H6A	109.5	C10—N5A—P1	120.7 (4)
C5—C6—H6A	109.5	C12—N5A—P1	113.6 (4)
N4—C6—H6B	109.5		
			
C5^i^—N3—C3—C4	58.56 (8)	C7—N5—C9—C10	−65.1 (5)
C5—N3—C3—C4	−58.56 (7)	C11—N5—C9—C10	53.2 (6)
P1—N3—C3—C4	180.000 (1)	N5—C9—C10—N5A	12.1 (2)
C6^i^—N4—C4—C3	−58.71 (8)	C7—N5—C11—C12	51.6 (5)
C6—N4—C4—C3	58.71 (8)	C9—N5—C11—C12	−66.5 (6)
N3—C3—C4—N4	0.000 (1)	N5—C11—C12—N5A	11.5 (2)
C3—N3—C5—C6	58.18 (13)	C7—C8—N5A—C10	−65.4 (4)
C5^i^—N3—C5—C6	−58.81 (16)	C7—C8—N5A—C12	49.2 (6)
P1—N3—C5—C6	−170.83 (9)	C7—C8—N5A—P1	168.13 (15)
C6^i^—N4—C6—C5	57.90 (15)	C9—C10—N5A—C8	50.3 (4)
C4—N4—C6—C5	−58.99 (13)	C9—C10—N5A—C12	−66.2 (5)
N3—C5—C6—N4	0.48 (15)	C9—C10—N5A—P1	162.7 (3)
C11—N5—C7—C8	−65.9 (4)	C11—C12—N5A—C8	−62.7 (6)
C9—N5—C7—C8	51.0 (5)	C11—C12—N5A—C10	53.0 (6)
N5—C7—C8—N5A	13.2 (3)	C11—C12—N5A—P1	−172.0 (2)

Symmetry code: (i) *x*, −*y*+3/2, *z*.

### Phosphanetricarbonitrile–1,4-diazabicyclo[2.2.2]octane (1/2) (2) Hydrogen-bond geometry (Å, º)

**Table:**

*D*—H···*A*	*D*—H	H···*A*	*D*···*A*	*D*—H···*A*
C8—H8*B*···N2^ii^	0.97 (2)	2.53 (2)	3.260 (2)	132 (2)

Symmetry code: (ii) *x*−1, *y*, *z*.
